# Effects of Nitrate Intake on Myocardial Ischemia-Reperfusion Injury in Diabetic Rats

**DOI:** 10.5935/abc.20160137

**Published:** 2016-10

**Authors:** Sajad Jeddi, Saeedeh Khalifi, Mahboubeh Ghanbari, Fatemeh Bageripour, Asghar Ghasemi

**Affiliations:** 1 Endocrine Physiology Research Center and Endocrine Research Center, Research Institute for Endocrine Sciences, Shahid Beheshti University of Medical Sciences, Tehran, Iran; 2 Department of Medical Laboratory Sciences, Faculty of Paramedical Sciences, Shahid Beheshti University of Medical Sciences, Tehran, Iran

**Keywords:** Coronary Artery Disease, Nitrates, Nitrites, Myocardial Reperfusion, Diabetes, Ischemia

## Abstract

**Background:**

Coronary artery disease is 2-3 times more common in diabetic individuals. Dietary nitrate/nitrite has beneficial effects in both diabetes and cardiovascular disease. It also has protective effects against myocardial ischemia-reperfusion (IR) injury in healthy animals. However, the effects of nitrate on myocardial IR injury in diabetic rats have not yet been investigated.

**Objective:**

We examined the effects of dietary nitrate on myocardial IR injury in streptozotocin-nicotinamide-induced diabetic rats.

**Method:**

Rats were divided into four groups (n=7 in each group): control, control+nitrate, diabetes, and diabetes+nitrate. Type 2 diabetes was induced by injection of streptozotocin and nicotinamide. Nitrate (sodium nitrate) was added to drinking water (100 mg/L) for 2 months. The hearts were perfused in a Langendorff apparatus at 2 months and assessed before (baseline) and after myocardial IR for the following parameters: left ventricular developed pressure (LVDP), minimum and maximum rates of pressure change in the left ventricle (±dP/dt), endothelial nitric oxide (NO) synthase (eNOS) and inducible NO synthase (iNOS) mRNA expression, and levels of malondialdehyde (MDA) and NO metabolites (NOx).

**Results:**

Recovery of LVDP and ±dP/dt was lower in diabetic rats versus controls, but almost normalized after nitrate intake. Diabetic rats had lower eNOS and higher iNOS expression both at baseline and after IR, and dietary nitrate restored these parameters to normal values after IR. Compared with controls, heart NOx level was lower in diabetic rats at baseline but was higher after IR. Diabetic rats had higher MDA levels both at baseline and after IR, which along with heart NOx levels decreased following nitrate intake.

**Conclusion:**

Dietary nitrate in diabetic rats provides cardioprotection against IR injury by regulating eNOS and iNOS expression and inhibiting lipid peroxidation in the heart.

## Introduction

The worldwide prevalence of diabetes has recently increased over twofold and is estimated to reach 592 million by 2035.^1^ Coronary artery disease is two to three times more common in diabetic patients and is the cause of mortality in over half of these individuals.^2^ Although various treatments are currently available for patients with diabetes, they lack sufficient efficacy; therefore, new strategies must be examined.^3^

Nitrate intake is considered a new potential strategy for managing type 2 diabetes.^3^ During the last half century, dietary nitrate has been considered unsafe due to its theoretical association with the development of diseases (including gastric cancer).^4^ This view is now being questioned after enzyme-independent production of nitric oxide (NO) from nitrate/nitrite has been found in tissues^5^ The nitrate/nitrite/NO pathway is a booster system for the L-arginine-NO synthase (NOs) pathway, mainly in conditions of NOs dysfunction^6,7^ Depending on the NOs isoform involved, NO could have protective or detrimental effects on the heart.^8^ Endothelial NOS (eNOS) is localized in caveolae where it controls heart rate, contraction, diastolic relaxation, and oxygen consumption. Inducible NOS (iNOS) is not present in healthy hearts but is expressed during pathological states, including ischemia and hyperglycemia, when it contributes to impair eNOS function and possibly worsen myocardial injury.^9-11^

Dietary nitrate reduces blood pressure, prevents endothelial dysfunction, and inhibits platelet aggregation.^12^ Recent investigations in animals indicate that dietary nitrate/nitrite has beneficial effects in diabetes and could increase pancreatic blood flow and insulin secretion,^13^ and improve both insulin resistance^14^ and glucose tolerance.^15^ Nitrate/nitrite has protective effects against myocardial ischemia-reperfusion (IR) injury in healthy animals.^6,16^ However, no studies have addressed to date the effects of nitrate on myocardial IR injury in diabetes. Therefore, the aim of this study was to determine the cardioprotective effects of nitrate on IR injury in streptozotocin (STZ)-nicotinamide (NA)-induced diabetic rats.

## Methods

The proposal of this study was approved by the Institutional Animal Care and Use Committee (IACUC) of the Research Institute for Endocrine Sciences (RIES, permit number: 12 EC RIES 92/10/25) at Shahid Beheshti University of Medical Sciences (Tehran, Iran).

### Animals

Male Wistar rats (2-month-old, 170-200 g) were obtained from the RIES laboratory animal house at Shahid Beheshti University of Medical Sciences. During the study, the animals were housed in an animal room with a temperature of 22 ± 2 °C and relative humidity of 50 ± 6%, with free access to standard rat chow (Pars Co., Tehran) and tap water. The animals were adapted to an inverse 12:12 h light-dark cycle for 2 weeks.

Diabetes was induced in the animals by intraperitoneal (IP) injection of NA (95 mg/kg) 15 min before an IP injection of STZ (65 mg/kg). Ten days after injection of STZ-NA, blood samples were obtained from a tail vein of the rats, and the animals with glucose levels > 126 mg/dL were considered diabetics.^17^ Rats were then allocated to four groups (n=7 each): control (C), control-nitrate (CN), diabetes (D), and diabetes-nitrate (DN). Each group was divided into two subgroups: pre-IR (baseline), and post-IR. Nitrate (sodium nitrate, Merck KGaA, 64271 Darmstadt, Germany) was added to the drinking water of the animals in the CN and DN groups at a concentration of 100 mg/L for 2 months.

### Measurements of hemodynamic parameters in Langendorff perfused hearts

At 2 months, all rats were anesthetized by IP injection of ketamine/xylazine (50 mg/kg and 10 mg/kg), and hearts from all groups were rapidly removed and immersed in ice-cold perfusion buffer. After cannulation of the aorta, the hearts were perfused in a Langendorff apparatus with a Krebs-Henseleit solution (containing [in mM] NaCl 118, NaHCO_3_ 25, KCl 4.7, MgCl_2_ 1.2, CaCl_2_ 2.5, KH_2_PO_4_ 1.2, glucose 11, and pH=7.4) under constant pressure (75 mmHg) and temperature of 37 ºC. Krebs solution was oxygenated with 95% O_2_ and 5% CO_2_. After 20 minutes of stabilization, the hearts of the animals in all groups were exposed to 30 minutes of global ischemia, followed by 60 minutes of reperfusion. A latex balloon was inserted into the left ventricle for measurement of hemodynamic parameters including left ventricular end diastolic pressure (LVEDP), left ventricular developed pressure (LVDP), and the minimum and maximum rates of pressure change in the left ventricle (±dP/dt). LVEDP, LVDP, and ±dP/dt were digitalized by a data acquisition system (Power Lab, AD Instrument, Australia). Post-ischemic hemodynamic parameters were assessed by the recovery of LVEDP, LVDP, and ±dP/dt.

### Assessment of lipid peroxidation

Levels of malondialdehyde (MDA), an indirect marker of cellular injury that reflects the extent of systemic lipid peroxidation in the heart, were measured by the method by Oshawa et al.^18^ In brief, heart samples were collected at baseline and during the post-IR period and then homogenized in phosphate buffered saline (PBS) (1:5, w/v) using a mini homogenizer (MICCRA-D1, Germany). Tissue homogenates were centrifuged (1,000 g, 4 ºC, 10 min) and supernatants were collected for measurement of MDA levels; 2.5 mL of trichloroacetic acid (20%) was added to 0.5 mL of supernatant followed by addition of 1 mL of thiobarbituric acid (0.67%). The mixture was placed in a water bath at 95 °C for 30 min and after cooling, 4 mL of n-butanol was added. The mixture was vortexed and after centrifugation at 1,100 g for 10 min, the absorbance of the upper layer was read at 532 nm using an ELISA reader (BioTek, Power wave XS2). The concentration of MDA was calculated using a standard curve of 1, 1, 3, 3-tetraethoxypropane and expressed as *µ*mol/L.

### Measurement of NO metabolites (NOx)

The NOx levels in the hearts were measured by the Griess method. In brief, heart samples collected at baseline and at the post-IR period were rinsed and homogenized in PBS (1:5, w/v), and centrifuged at 15,000 g for 20 min. Supernatant was deproteinized by adding zinc sulfate (15 mg/mL); 100 *µ*L of the supernatant were transferred to a microplate well and 100 *µ*L of vanadium (III) chloride (8 mg/mL) were added to each well to reduce nitrate to nitrite; 50 *µ*L of sulfanilamide (2%) and 50 *µ*L of N-1-(naphthyl) ethylenediamine (0.1%) were then added, and samples were incubated for 30 min at 37 °C; absorbance was read at 540 nm using the ELISA reader. NOx concentration was determined from a linear standard curve established by 0-50 *µ*mol/L sodium nitrate. Tissue NOx levels were expressed as *µ*mol/L.

### Assessment of myocardial injury markers in coronary flow (CF)

At the start of reperfusion, CF was collected for a period of 5 minutes to measure myocardial enzyme leakage (myocardial injury markers), including creatine kinase MB (CK-MB) and lactate dehydrogenase (LDH). Levels of CK-MB and LDH in CF were measured using commercial kits (Pars Azmoon, Tehran, Iran).

Intra-assay coefficients of variation for NOx, CK-MB, and LDH measurements were 3.7%, 4.1%, and 4.7%, respectively.

### RNA extraction, cDNA synthesis, and real-time PCR

In all groups, samples from the left heart ventricle were obtained at baseline and during the post-IR period for RNA extraction using a standard, sterile surgical method. Total RNA was extracted from heart tissue using an RNX-Plus solution kit (Fermentase, Cinagen Co., Iran), according to the manufacturer's description and treated with RNase-free DNase to remove any residual genomic DNA. RNA quantity and purity were measured using the NanoDrop 1000 (Thermo Scientific, Waltham, MA, USA); cDNAs were synthesized by incubating total RNA with RevertAid H Minus Reverse Transcriptase, DNase I, Random Hexamer Primer, dNTPs, and RiboLock RNase Inhibitor, for 10 min at 25 °C, followed by 60 min at 42 °C in a final volume of 20 *µ*L. The reaction was terminated by heating at 70 °C for 5 min.

Quantitative real-time PCR was performed with Rotor-Gene 6000 (Corbett Life Science, Corbett Research, Sydney, Australia). Reactions were performed in 25 *µ*L aliquots containing 12.5 *µ*L SYBR Green PCR Master Mix (Jena Bioscience, Germany), 1 *µ*L forward primer, 1 *µ*L reverse primer, 8.5 *µ*L RNase- free water, and 2 *µ*L of the cDNA. The primer sequences used for real-time (RT)-PCR were forward 5′-TACAGCTTCACCACCACAGC-3′ and reverse 5′-CACACTGCTAGAGGTGCTGGAA-3′ for beta-actin; forward 5′-ATGCCACAGGATTCCATACC-3′ and reverse 5′-TGTGCTGGCTTTGGTGAGGTTTGA-3′ for eNOS, and forward 5′-TGGCCTCCCTCTGGAAAGA-3′ and reverse 5′-TGCTGAGCTGACAGAGTAGTA-3′ for iNOS. PCR amplifications were performed by the three following cycle programs: (1) denaturation of cDNA (1 cycle: 10 min at 95 °C), (2) amplification (40 cycles: 15 sec at 95 °C, 30 sec at 60 °C, and 30 sec at 72 °C), (3) melting curve analysis (1 cycle: 72 to 95 °C with temperature transition rate 1 °C/sec for 5 sec). Real-time quantification was monitored by measuring the fluorescence activity. To compare the groups, the mRNA levels of eNOS and iNOS were measured as relative expression using 2^−ΔΔCT^ values and normalized to beta-actin generated from the same sample; where ΔΔCT = [CT _iNOS or eNOS (case)_ − CT _beta-actin (case)_] − [CT _iNOS or eNOS (control)_ − CT _beta-actin (control)_].^19^ The specificity of the PCR reactions was verified by melting curve analysis.

### Statistical analysis

All values are expressed as mean ± standard error of the mean (SEM). The statistical analysis was performed using SPSS software, version 20 (SPSS, Chicago, IL, USA). The Kolmogorov-Smirnov and Shapiro-Wilk tests were used to check the normality of the study data, with p-values > 0.05 indicating normal distribution. Parametric and nonparametric tests were used for analysis of data with normal and non-normal distribution, respectively.^20^ Repeated measurement analysis of variance (ANOVA) was used to compare the hemodynamic parameters (LVDP, LVEDP, and ±dP/dt) at different time points. Student's sample *t* test was used to compare MDA and NOx levels at baseline and post-IR in each group. One-way ANOVA with Tukey *post-hoc* test was used for comparison among different groups of levels of MDA, NOx, CK-MB, and LDH. The Mann-Whitney U test was used to compare gene expression in different groups. Two-sided p-values < 0.05 were considered statistically significant.

## Results

The initial blood glucose levels, and body and heart weights of the animals were similar in all groups. After STZ-NA injection and compared with controls, diabetic rats had increased blood glucose levels (196.8 ± 26.4 *versus* 82.6 ± 4.5 mg/dL; p < 0.05), decreased body weight (239.7 ± 12.3 *versus* 331.7 ± 13.7 g; p < 0.05), and significantly increased heart weight to body weight ratio (0.48 ± 0.01 *versus* 0.37 ± 0.01%). At the end of the study (day 70), the area under the curve of the plasma glucose concentration in diabetic rats (20,264 ± 659 mg/dL/60 min) during an intravenous glucose tolerance test (GTT) was significantly (p < 0.05) higher when compared with that in control rats (7,825 ± 247 mg/dL/60 min, p < 0.05).

**Figure 1:**
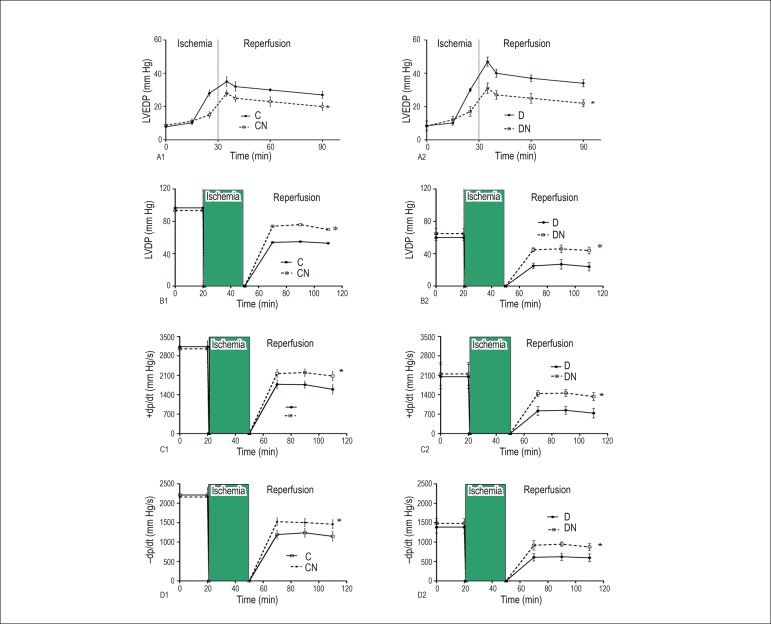
Recovery of cardiac function after IR injury. A) Left ventricular end diastolic pressure (LVEDP); B) Left ventricular developed pressure (LVDP); C) Maximum rates of pressure change in the left ventricle (+dP/dt); D. Minimum rates of pressure change in the left ventricle (-dP/dt). Values are mean ± SEM; (n = 7 in each group); *p < 0.05, as compared with the control and diabetic gr oups. Control (C), control-nitrate (CN), diabetes (D), and diabetes-nitrate (DN).

The effects of dietary nitrate on hemodynamic parameters in isolated hearts during the stabilization and IR period are shown in Table 1 and Figure 1. During the stabilization period, hearts from diabetic rats had significantly lower baseline LVDP and ±dP/dt values (p < 0.05) as compared with control rats. Nitrate intake had no effect on baseline LVDP and ±dP/dt values in heart samples from CN and DN rats compared with the values in control and diabetic rats, respectively (Table 1).

**Table 1:** Parameters of cardiac function during the stabilization period

	Control	Control-Nitrate	Diabetes	Diabetes-Nitrate
LVEDP (mmHg)	8.5 ± 2.2	8.8 ± 2.8	8.2 ± 1.8	7.9 ± 2.3
LVDP (mmHg)	96.7 ± 7.6	93.4 ± 6.6	60 ± 3.9*	65 ± 6.3*
+dP/dt (mmHg/s)	3135 ± 211	3055 ± 247	2052 ± 434*	2152 ± 412*
–dP/dt (mmHg/s)	2215 ± 188	2163 ± 205	1387 ± 154*	1484 ± 123*

Data are represented as mean ± SEM.  LVEDP: left ventricular end diastolic pressure; LVDP: left ventricular developed pressure, and minimum and maximum rates of pressure change in the left ventricle (± dP/dt)

* p < 0.05 statistically  significant deference between  group D versus C group and group DN versus CN. Control (C), control-nitrate (CN), diabetes (D), and diabetes-nitrate (DN).

In all groups, LVEDP gradually increased during the 30 minutes of ischemia. However, diabetic rats compared with control ones displayed a significant increase in LVEDP (p < 0.05), while CN and DN rats showed a significant decrease (p < 0.01) in this parameter when compared with control and diabetic rats, respectively. Nitrate intake improved diastolic properties, as indicated by a lower LVEDP during reperfusion in heart samples of rats in the CN and DN groups and prevented a hypercontractile response during the early phase of reperfusion (Figure 1-A).

**Figure 2:**
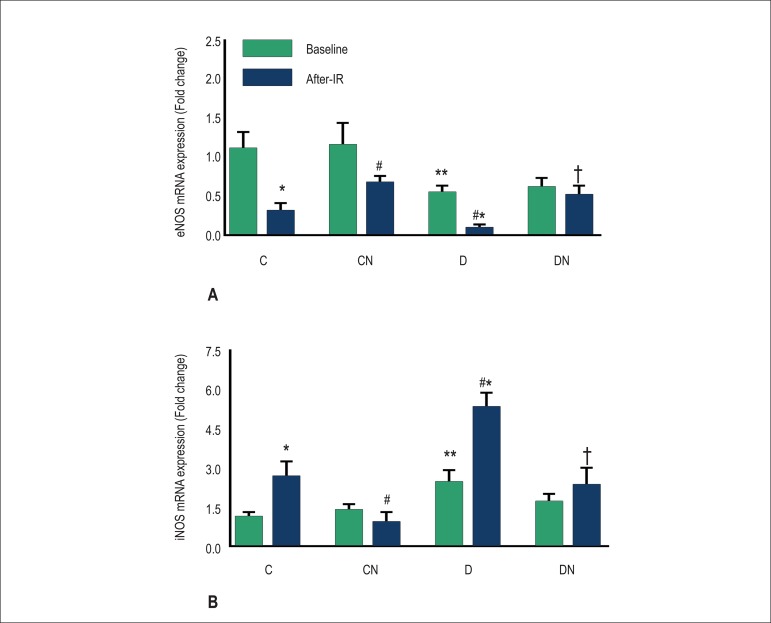
Effect of nitrate intake on eNOS (A) and iNOS (B) mRNA expression. Values are expressed as mean ± SEM; *p < 0.05, comparing the pre-IR with the post-IR period in each group. **p < 0.05 comparing the pre-IR period in each group with controls. #p < 0.05 comparing the post-IR period in each group with controls. †p < 0.05 comparing the post-IR period between the DN and D groups. Control (C), control-nitrate (CN), diabetes (D), and diabetes-nitrate (DN).

Compared with controls, STZ-NA-induced diabetic rats had significantly lower postischemic LVDP and ±dP/dt values. Nitrate intake restored the decreased LVDP and ±dP/dt values to near preischemic values in both the CN and DN groups, compared with the control and diabetic groups, respectively (p < 0.01) (Figure 1-B, C, D). Compared with baseline values, expression of eNOS decreased significantly and that of iNOS increased significantly in the control group after IR. Decreased eNOS expression and increased iNOS expression were observed in hearts from diabetic rats when compared with controls both before and after IR; dietary nitrate restored eNOS and iNOS expression to near normal values after IR in the CN and DN groups. Nitrate intake had no effect on baseline eNOS and iNOS expression in hearts of rats in the CN and DN groups compared with those in the control and diabetic groups, respectively (Figure 2).

**Figure 3:**
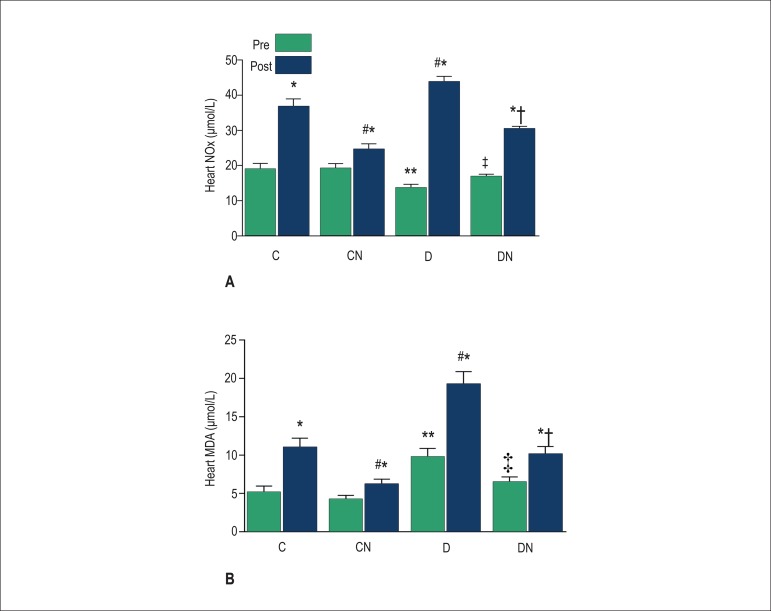
Changes in NOx (A) and MDA (B) levels in the hearts of rats in different groups. Values are expressed as mean ± SEM; (n = 7 in each group); *p < 0.05 comparing the pre-IR with the post-IR period in each group. **p < 0.05 comparing the pre-IR period in each group with controls. #p < 0.05 comparing the post-IR period in each group with controls. ‡p < 0.05 comparing the pre-IR period in the DN and D groups. †p < 0.05 comparing the post-IR period between the DN and D groups. Control (C), control-nitrate (CN), diabetes (D), and diabetes-nitrate.

In all groups, heart NOx levels increased significantly (p < 0.05) after IR when compared with baseline values. Dietary nitrate had no effect on baseline heart NOx levels in the CN group, which showed comparable levels to those in the control group. After ischemia, heart NOx levels increased in the control group but not in the CN group (p < 0.05), suggesting a protective effect of dietary nitrate. Compared with controls, heart NOx level was lower in diabetic rats before IR, but was higher after IR and decreased following nitrate intake in the DN group, both before and after IR when compared with diabetic rats (Figure 3-A).

**Figure 4:**
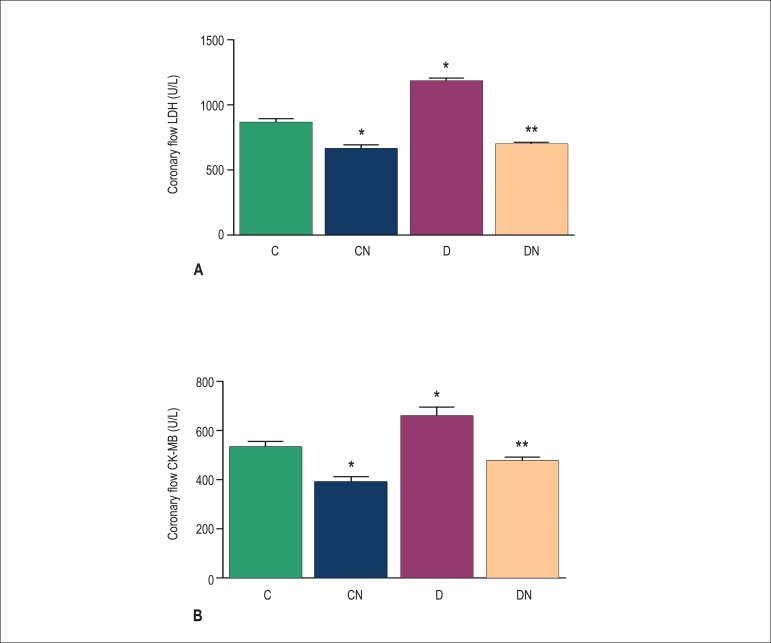
Effect of nitrate intake on levels of LDH (A) and CK-MB (B) in coronary flow 5 min after reperfusion. Values are expressed as mean ± SEM; (n = 7 in each group); *p < 0.05 compared with the control group. **p < 0.05 comparing the DN with the D group. Control (C), control-nitrate (CN), diabetes (D), and diabetes-nitrate (DN).

In all groups, heart MDA levels increased significantly (p < 0.05) after ischemia when compared with baseline values. Compared with controls, diabetic rats had significantly higher MDA levels, both before and after IR (p < 0.05); nitrate intake restored the elevated MDA levels to near normal values in the CN and DN groups as compared with levels in the control and diabetic group, respectively (Figure 3-B). Compared with the control group, CK-MB and LDH levels were significantly higher (p < 0.05) in the diabetic group (p < 0.05), and dietary nitrate significantly reduced the release of CK-MB and LDH in the CF of rats in the CN and DN groups *versus* the control and diabetic groups, respectively (Figure 4).

## Discussion

Nitrate intake improved the recovery of the cardiac function after ischemia in both normal and diabetic rats. This cardioprotective effect was associated with prevention of increased iNOS and decreased eNOS mRNA expression in heart tissue. In addition, nitrate intake restored heart levels of both NO_x_ and MDA to normal values.

In this study, we showed for the first time that chronic intake of a low dose of nitrate (100 mg/L for 2 months) protected the heart of rats in the control group from IR injury. In humans, a similar dose can be achieved with a vegetable-rich diet. Most studies assessing the effects of nitrate on IR injury have been performed *in vitro*.^21-23^ Webb et al.^21^ have reported that nitrite infusion in rats (0.7-7 mg/L) during ischemia in the Langendorff apparatus reduced infarct size and improved left ventricular function.^21^ An additional investigation by Gonzalez et al.^23^ has documented a similar protection in an *in vivo* canine model.^23^ Duraunski et al.^22^ have also shown that administration of nitrite (48 nmoL) during ischemia in mice reduced the infarct size and protected the heart from IR injury.^22^ Bryan et al.,^24^ in an *in vivo* study, assessed the effects of short-term nitrate intake (1,000 mg/L in drinking water for 7 days) on IR injury and showed that nitrate reduced the infarct size in response to a myocardial IR injury.^24^ However, it remains disputable whether conclusions from a short-term study could be extended to a long-term situation.^25^ Therefore, we assessed the effects of chronic low-dose nitrate intake on IR injury in STZ-NA-induced diabetic rats. This is a suitable model for assessment of the effectiveness of new potential antidiabetic agents and has been reported to be close to human type 2 diabetes. However, STZ-NA-induced diabetic rats, as a model for nonobese type 2 diabetes, do not display insulin resistance, which is the main characteristic of type 2 diabetes.^15,17^ In this study, we defined diabetes using a blood glucose cutoff level of 126 mg/dL because the STZ-NA model of type 2 diabetes is associated with moderate hyperglycemia.^17^ This value, when determined during fasting, is adopted in human studies to define diabetes.^26^ In addition, the occurrence of an abnormal GTT indicates that diabetes was successfully induced.

In this study, hearts from STZ-NA-induced diabetic rats had lower baseline cardiac functions, a controversial finding in different animal models of diabetes.^27-29^ Lower recovery of cardiac function has been observed in diabetic rats following IR when compared with controls. The effects of type 2 diabetes on myocardial IR injury in animal studies are a controversial issue, and both higher^30^ and lower^30^ sensitivity to injury or even no effect^31^ have been reported. These inconsistent results can be partially explained by the severity and duration of type 2 diabetes and also the change in metabolic profiles following induction of diabetes.^28,32^ Moreover, the differences between models of type 2 diabetes, the degree of IR injury, and the species of the rat with type 2 diabetes can modify the baseline cardiac functions and susceptibility to myocardial IR injury.^28^

Our results show that diabetic rats had lower eNOS and higher iNOS expression both at baseline and after IR. Heart NOx level was also lower before IR, but was higher after IR, and heart MDA level was higher both before and after IR. Previous studies have shown that although iNOS-induced NO formation increases NO levels in diabetes, elevation in reactive oxygen species (ROS), while simultaneously interacting with NO, lead to decreased NO bioavailability in baseline serum and heart. In addition, NO may rapidly be scavenged by free radicals and produce peroxynitrite, leading to baseline myocardial injury.^9,11^ Also, a marked increase in production of ROS and decrease in antioxidant capacity in hyperglycemic conditions lead to the development of oxidative stress, lipid peroxidation, and cell membrane injury, which may cause leakage of LDH and CK-MB, suggesting an increased oxidative stress and cell necrosis in STZ-NA-induced diabetes.^11^

To the best of our knowledge, no study so far has documented the effect of nitrate intake on IR injury in STZ-NA-induced diabetic rats. We have previously shown that dietary nitrate prevents an increase in systolic blood pressure and serum glucose, improves glucose tolerance, and restores dyslipidemia in STZ-NA-induced diabetic rats.^15^ Jiang et al.^33^ have reported that dietary nitrite (50 mg/L for 4 weeks) in db/db diabetic mice improves insulin signaling through GLUT4 translocation to the membrane.^33^ Ohtake et al. have also shown that dietary nitrite (150 mg/L for 10 weeks) improves insulin resistance in type 2 diabetic mice.^14^

Nitrate intake in this study also offered cardioprotection by decreasing the levels of myocardial injury markers in CF and lipid peroxidation levels in the hearts of diabetic rats after IR, an effect that may be related to the antioxidative properties of nitrate intake that protect the membrane of heart cells by inhibiting lipid peroxidation and decreasing the leakage of cytosolic enzymes.^11^ We have also previously reported that chronic treatment with a low nitrate dose attenuates oxidative stress in STZ-NA-induced diabetic rats by increasing serum total antioxidant capacity and catalase activity.^15^

In the present study, we found that nitrate intake protected the heart from IR injury by restoring iNOS and eNOS expression to normal values and subsequently reducing the accumulation of NO after IR. Previous studies have reported that nitrite intake improves insulin signaling^34^ and increases insulin secretion in rats.^13^ Insulin activates Akt and increases eNOS activity through the PI3K-Akt-eNOS pathway,^34,35^ which activates eNOS through phosphorylation of serine 1177^35^ and increased NO production. In addition, insulin decreases iNOS-induced NO production and reduces IR-induced peroxynitrite formation.^36^ Increased eNOS expression and decreased iNOS expression by insulin are both cardioprotective. This gives us a basis to hypothesize that nitrate intake protects the heart by increasing eNOS expression and decreasing iNOS expression via a decrease in oxidative stress and an increase in insulin secretion.

Our results show that nitrate intake was insufficient to restore basal hemodynamic function to near normal values in diabetic rats, because in the diabetic state, before NO acts it is inactivated by ROS. Evidence shows that eNOS may be uncoupled in diabetic conditions due to a decrease in tetrahydrobiopterin, a NOS cofactor that increases superoxide anion formation and impairs eNOS function in the heart. Moreover, iNOS may have important chronic deleterious effects, *i.e*., irreversible impairment of basal contractile function mediated via peroxynitrite that is not readily reversible by nitrate intake; hence, other treatments such as radical scavengers seem essential.^37^

Some limitations of our study should be considered when the results are interpreted. First, we did not measure the levels of eNOS and iNOS proteins. Although mRNA changes do not necessarily reflect protein changes, the mRNA expression is still informative.^38^ Second, we did not use a pharmacological approach to confirm our results regarding decreased iNOS expression and increased eNOS expression after nitrate intake, since it has been shown that both deletion of iNOS gene and inhibition of iNOS could provide cardioprotection in diabetic animals.^39^

## Conclusion

Nitrate intake restored cardiac function to near preischemic values after IR in diabetic rats by blocking the pathological increases in iNOS expression, as well as the pathological decrease in eNOS expression. It also restored NOx and MDA levels in the heart to normal values both before and after ischemia.
